# Poison for Arrow Tips

**Published:** 1890-07

**Authors:** 


### POISON FOR ARROW TIPS.

### HOW THE PIUTE INDIANS PREPARED THE DEADLY PASTE.

We are indebted to Mr. Frank Smith, of Whitewater, for a very graphic account of the manner in which a Piute Indian prepared his deadly arrows. He gathered a dozen or more rattlesnake heads, and put them in a spherical earthen vessel. With these he put half a pint of a species of large red ant, that is found hereabouts. The bite of this ant is more poisonous than that of a bee. Upon these he poured a bit of water, and then sealed up with moist earth and a lid this vessel. He then dug a hole two feet deep into the ground, in which he built a roaring fire, and put in some stones. When the interior of the hole and stones were red hot, he made a place in the bottom for the earthen vessel, and put it in. About it and upon it he put the coals and hot stones, and upon the top he built a fierce fire, and kept it up for twenty-four hours. Then he dug out his vessel, and, standing off with a long pole, he disengaged the top and let the fumes escape. He insisted that had they struck his face, it would have killed him. The mass left in the vessel was a dark brown paste.

To test the efficacy of his concoction, the Indian with his hunting knife made a cut in his bare leg, just below the ankle. Then taking a stick, he dipped it into the poison, and touched the descending blood at the ankle. It immediately began to sizzle, as if it were cooking the blood, and the poison followed the blood right on up the leg, sizzling its way until the Indian scraped it off with the knife. He assured our informant that had he allowed it to reach the mouth of the wound he would have been a dead man.

